# The Anodyne Treatment in Croup

**Published:** 1848

**Authors:** 


### ARTICLE VIII.

### *The Anodyne Treatment in Croup.*

Everything relating to the treatment of so serious a malady as croup, is of interest to the profession. The following, from the Transactions of the Philadelphia College of Physicians, shows a successful result of treatment in the hands of an eminent and experienced practitioner.

"Dr. Parish related the history of a case of membranous croup of a severe character, and attended with all the symptoms of approaching death, which recently recovered under his care, without an operation for tracheotomy.

"The patient was a child of 18 months old, which was attacked with what was supposed to be an ordinary catarrh, attended with a harsh, dry cough. Simple domestic remedies had been admnistered for several days without effect, before the doctor was sent for.

"When the patient was seen by him, the cough was 'croupy,' and the breathing obstructed to an alarming extent. On looking into the fauces, the whole back part of the throat was found lined with a thick, tenacious secretion. An emetic of two grains of turpeth mineral was immediately administered, which operated promptly, but without relief. Calomel, grs. v. was administered, to be followed by castor oil in a few hours. After the operation of the purgative, there was still no decided relief; the turpeth mineral emetic was continued regularly every four hours, and the calomel in small doses every two hours, for several days; but the disease steadily progressed, no discharge of membrane having been induced; the breathing became more distressing, and finally, the child was unable to cry; the cough was dry, and less developed, and the bronchial tubes appeared to be rapidly filling up. At this stage of the complaint, injections of assafœtida and laudanum were given every four hours, solely with a view of assuaging the sufferings of the child, and all other medicines were suspended. The full effect of the opium was induced, and the jactitation and restlessness diminished, though the breathing continued as bad as ever. The child lay upon the pillow with the head thrown back, and was several times supposed to be dying, from the violence of the paroxysms of dyspnœa. The doctor left late in the evening, with directons to continue the anodyne, expecting to find his patient dead in the morning. On the morning visit, he was surprised to find that the paroxysms of extreme difficulty of breathing, had been less frequent, and that the child had slept with comparative comfort, though the respiration was still exceedingly laborious. It was found that a discharge of thick yellow mucus had begun to isue from the nostrils during the night, and on examining the throat, it was evident that the membrane lining the fauces was loosing. The bowels had also been freely moved with copious yellow dejections.

"This state of things afforded encouragement to resume the use of the turpeth mineral, which acted promptly, bringing away large quantities of thick, yellow mucus, to the great relief of the infant, who, from this time, went on improving, and recovered rapidly.

"Dr. P. had no expectation of accomplishing any permanent good by the use of the anodyne in this case 'believing that the mechanical obstruction of the trachea and bronchial tubes must inevitably destroy life. It becomes a question, however, how far the dyspnœa in cases of membranous croup, may be the result of nervous spasm, as well as of a mechanical impediment to the passage of air into the lungs. It is evident that the dyspnœa is, to a certain extent, paroxysmal, and it is also true, that in many cases where death has occurred, the accumulation of membranous deposit in the air passages, as discovered on a *post-mortem* examination, has not been sufficient to produce strangulation from mere mechanical obstruction. By keeping, therefore, the system under the influence of antispasmodics and anodynes, in addition to remedies calculated to arrest the inflammation, may we not gain time, and enable the latter to have their full effect in arresting the disease, and in producing softening of the membranous deposit?

"The doctor believed that in the management of this intractable malady, we have neglected too much the use of this class of remedies. He would also take this occasion to express his satisfaction with the action of turpeth minera as an emetic in croup. He had used it on several occasions, since it had been so warmly recommended to the college in the communication of Dr. Hubbard, of Maine, and had been highly pleased with it. It acts promptly and powerfully, without leaving behind it the depressing effects of the antimonials.

"The emetic may be repeated at short intervals, and continued as in this case, for many hours, without the risk of alarming depression."

